# Skilled attendant at birth and newborn survival in Sub–Saharan Africa

**DOI:** 10.7189/jogh.07.020504

**Published:** 2017-12

**Authors:** Agbessi Amouzou, Meng Ziqi, Liliana Carvajal–Aguirre, John Quinley

**Affiliations:** 1 Institute for International Programs, Department of International Health, Johns Hopkins Bloomberg School of Public Health, Baltimore, USA; 2 Data and Analytics, Division of Data, Research and Policy, UNICEF, New York, New York, USA

## Abstract

**Background:**

Recent studies have shown higher neonatal mortality among births delivered by a skilled attendant at birth (SAB) compared to those who were not in sub–Saharan African countries. Deaths during the neonatal period are concentrated in the first 7 days of life, with about one third of these deaths occurring during the first day of life. We reassessed the relationship between SAB and neonatal mortality by distinguishing deaths on the first day of life from those on days 2–27.

**Methods:**

We used data on births in the past five years from recent demographic and health survey (DHS) between 2010 and 2014 in 20 countries in sub–Saharan Africa. The main categorical outcome was 1) newborns who died within the first day of birth (day 0–1), 2) newborns who died between days 2–27, and 3) newborns who survived the neonatal period. We ran generalized linear mixed model with multinomial distribution and random effect for country on pooled data. Additionally, we ran a separate model restricted to births with SAB and assessed the association of receipt of seven antenatal care (ANC) and two immediate postnatal care interventions on risk of death on days 0–1 and days 2–27. These variables were assessed as proxy of quality of antenatal and postnatal care.

**Results:**

We found no statistically significant difference in risk of death on first day of life between newborns with SAB compared to those without. However, after the first day of life, newborns delivered with SAB were 16% less likely to die within 2–27 days than those without SAB (OR = 0.84, 95% CI = 0.71–0.99). Among births with skilled attendant, those who were weighed at birth and those who were initiated early on breastfeeding were significantly less likely to die on days 0–1 (respectively OR = 0.42 95% CI = 0.29–0.62 and OR = 0.24, 95% CI 0.18–0.31) or on days 2–27 (OR = 0.60, 95% CI = 0.45–0.81 and OR = 0.59, 95% CI = 47–0.74, respectively). Newborns whose mothers received an additional ANC intervention had no improved survival chances during days 0–1 of life. However, there was significant association on days 2–27 where newborns whose mothers received an additional ANC interventions had higher survival chances (OR = 0.95, 95% CI = 0.93–0.98).

**Conclusion:**

Findings demonstrate the vulnerability of newborns immediately after birth, compounded with insufficient quality of care. Improving the quality of care around the time of birth will significantly improve survival and therefore accelerate reduction in neonatal mortality in sub–Saharan African countries. Improved approaches for measuring skilled attendant at birth are also needed.

Global level of mortality among children under–five has been halved since 1990, with a decline from 91 deaths per 1000 live birth to 43 in 2015. A similar decline was also observed in sub–Saharan Africa, the region with the highest burden of mortality [1]. The pace of mortality decline was much slower among neonates, with sub–Sahara Africa recording one of the slowest declines of 38% over the same period, just behind Oceania where level of mortality is much lower. Subsequently, there is an increasing share of newborn deaths among all under–five deaths, reaching over a third in sub–Saharan Africa. The increasing mortality compression to the first days of life has raised calls for greater focus on newborn, with the adoption of the Every Newborn Action Plan (ENAP) in June 2014 and the subsequent publication of a Lancet Newborn Series to galvanize evidence–based programming that would accelerate reduction of newborn death toward ending preventable deaths [2]. The ENAP highlighted the strategic benefit of focusing on quality care around the time of birth by ensuring that all pregnancies have access to skilled quality care necessary for a healthy pregnancy and to protect the life of the newborn, and care for small and sick newborns [3,4]. The WHO has defined skilled attendant at birth as “an accredited health professional — such as a midwife, doctor or nurse — who has been educated and trained to proficiency in the skills needed to manage normal (uncomplicated) pregnancies, childbirth and the immediate postnatal period, and in the identification, management and referral of complications in women and newborns” [5]. This definition is currently being revised to clarify the competencies and extend to the notion of competent qualified maternal and newborn health care professional. [6] However, it does not address the limitation in measurement of skilled attendant at birth. In the absence of comparable data on quality skilled care, so far the world has mostly relied on the indicator of access to skilled attendant at delivery to monitor the likelihood that pregnancies receive some sort of quality of delivery care. Despite its limitation, this indicator has been one of the key coverage monitoring indicator in the Millennium Development Goals and more recently also adopted in the Sustainable Development Goals [7]. Skilled attendant at birth is also monitored as a core indicator in the ENAP and the Ending Preventable Maternal Mortality (EPMM) [8]. However, increasing number of studies calls for going beyond monitoring simple contact with a health system to include content and quality of interventions received [9–11]. Furthermore, assessment of the association between births reported to have been delivered with skilled attendant and chances of survival beyond the neonatal period did not generate expected results, especially in sub–Saharan Africa and Asia. In a recent study that included three countries each of three regions – Asia, sub–Saharan Africa and Latin America, Singh and her colleagues showed that skilled delivery did not appear to improve the survival of the newborn on the first day or week of life in the sub–Saharan African and Asian countries [12]. Possible explanations to this counter–intuitive finding highlight low quality of maternal and newborn services such that, although pregnant women come into contact with the health system to deliver, critical interventions needed to save the newborn in case of complications during delivery or postnatal period are not always available [13,14]. Other explanations include selection bias and uncertainty in the measurement of skilled attendant at birth in household surveys. Most women respondents in these surveys are of low schooling and not able to recall the type of cadre of health worker that provided the delivery care [15]. Regarding the selection bias, it is thought that in resource–constrained settings where access to health facility remains challenging and coverage of health facility use relatively low, women accessing delivery services in health facilities are likely to be those of higher potential risk of obstetric complication. A substantial portion of these women arrive late in health facilities, which may also not be properly equipped to promptly attend to the emergency [16–18].

In this study, we reassessed this relationship in a larger number of sub–Saharan African countries and by distinguishing deaths on the first day of life from days 2–27 using a multinomial mixed model. We conjecture that if the selection effect is real, the positive association between SAB and mortality will be seen only during the first day of life, when newborns are particularly vulnerable. Past this period, a negative association should be observed. Regarding quality of care, we also assessed association between the receipt of a series of seven antenatal care and two immediate postnatal care interventions by women and their newborns and mortality on days 0–1 and days 2–27.

## DATA AND METHODS

We used data from recent Demographic and Health Surveys (DHSs), conducted in sub–Saharan Africa between 2010 and 2014, with information on child mortality collected using full birth history from women aged 15–49 and selected antenatal and postnatal interventions. Data were available for 20 countries in sub–Saharan Africa. **Table 1** includes the list of the countries. DHSs are USAID–funded nationally representative household surveys carried out about every five years in low– and middle–income countries [19]. The survey program started in the mid–1980s and has been a major source of demographic, reproductive and health data in these countries. Data are collected using typically a two–stage cluster sampling (with some variation in some countries), with first stage represented by population census enumeration areas, and the second stage by households. All women of reproductive age (15–49 years) in each sampled household are interviewed. Data are collected on several modules, including a full birth history module that captures information on every live birth a woman respondent ever had and the survival status of these births. For children who died, information is collected on age at death. The breakdown of the age at death depends on the age range. For deaths under one month, age at death is collected in days, starting from day 0 (as day of birth). For deaths over 1 month of age but under two–years, age at death is collected in months, and for deaths over two years, age at death is collected in years. This information is used to estimate mortality among children under–five (neonatal, post–neonatal, infant, under–five mortality). Another module includes information on health care provided during pregnancy, delivery and the postpartum period for all live births in the five years preceding the survey. This module captures data on antenatal care, assistance at delivery and postnatal care. A limited number of interventions delivered during these stages is also collected from women’s recall. The module allows computation of births who had a skilled attendant at birth. Skilled birth attendant is captured generally as doctor, nurse, and midwife but there are slight variations across countries with addition of special cadres considered skilled (**Table 1**). Linking this module to the full birth history module allows an analysis of the association between receipt of skilled delivery and neonatal mortality. We based the analysis on children born in the past five years preceding each survey. For the twenty countries with available data, these range from 1251 to 12 272 births for a total of 84 168 births.

**Table 1:** Percentage of births with skilled health personnel by country

Country	Survey year	Percentage of births with skilled health personnel	Neonatal mortality (five year preceding the survey)*	Number of live births in the five years preceding the survey	Definition of skilled attendant at birth
Benin	2011–2012	80.9	23	5147	Doctor, nurse/midwife
Burkina Faso	2010	65.9	28	5790	Doctor, nurse/midwife, auxiliary midwife
Burundi	2010	60.3	31	3007	Doctor, nurse/midwife
Cameroon	2011	63.6	31	4496	Doctor, nurse/midwife
Comoros	2012	82.2	24	1251	Doctor, nurse/midwife
Congo	2011–2012	92.5	22	3625	Doctor, nurse/midwife, Assistant
Cote D’Ivoire	2011–2012	59.4	38	3041	Doctor, nurse/midwife
Democratic Republic of the Congo	2013	80.1	28	7209	Doctor, nurse/midwife
Gabon	2012	89.3	26	2443	Doctor, nurse/midwife
Guinea	2012	45.3	33	2763	Doctor, nurse/midwife, auxiliary midwife
Liberia	2013	61.1	26	2984	Doctor, nurse/midwife
Mozambique	2011	54.3	30	4543	Doctor, nurse/midwife
Niger	2012	29.3	24	4738	Doctor, nurse/midwife
Nigeria	2013	38.1	37	12272	Doctor, nurse/midwife
Rwanda	2010	69.0	27	3119	Doctor, nurse/midwife
Senegal	2010–2011	65.1	29	4771	Doctor, nurse/midwife
Sierra Leone	2013	59.7	39	4652	Doctor, nurse/midwife
Uganda	2011	57.4	27	2949	Doctor, nurse/midwife
United Republic of Tanzania	2010	48.9	26	3010	Doctor/assistant medical officer, nurse/midwife, clinical officer, assistant clinical officer
Zimbabwe	2010–2011	66.2	31	2358	Doctor, nurse/midwife
**MEDIAN**		**62.3**			
**Correlation between SAB and NMR**		**-0.42 (*P* < 0.0619)**			

*From STATCompiler, StatCompiler.com, accessed on February 2, 2017.

### Variables

The main outcome is death during the neonatal period. We created three categories: 1) newborns who died on days 0–1, 2) newborns who died between days 2 and 27, and 3) children under–five who survived the neonatal period. We initially also separated out deaths on days 2–7 but results of preliminary analysis were similar to those of deaths on days 8–27. We therefore grouped them together.

In addition to a binary variable on whether a birth was assisted by a skilled attendant at birth or not, we considered an additional main independent variable related to interventions received by the mother during antenatal care to capture quality of ANC. These interventions include urine test, blood test, blood pressure measured, iron supplementation, tetanus protection at birth, counselled on pregnancy complications, tested for HIV and received results. We created a categorical variable of number of interventions received by summing the indicator variable representing each intervention. We use this composite indicator as a proxy for quality of care received by the women during their pregnancy. For postnatal interventions, we considered two immediate postnatal interventions: whether the newborn was weighed at birth and whether the newborn was initiated early on breastfeeding. The latter was captured by asking the mother whether the newborn was breastfed within one hour following birth. We could not consider other available postnatal indicators in the analysis due to possible selection bias that would be introduced, given newborns who died immediately during the first days of life would not have the same exposure time to the chance of receiving these interventions.

We considered as control variables, socio–economic and demographic variables with known effects on mortality. These included residence (urban, rural), wealth quintile (poorest, poorer, middle, richer, richest), marital status (single, married, other), parity (1, 2–4, 5 or more), mother’s age at birth (15–19, 20–29, 30–39, 40–49 years), and mother’s education level (no schooling, primary, secondary or higher).

### Analysis

We first described the coverage of skilled attendant at birth in the twenty countries. For each country, we computed and compared neonatal mortality rate separately for births with a skilled birth attendant and those without. We computed these rates on the three years preceding each survey using a life table approach [20]. To compare the rates, we computed 95% confidence intervals using Jackknife approach [21]. We then pooled all country data and fit a generalized linear mixed model with multinomial distribution and random effect for country. We used the third category of the outcome variables (children under–five who survived the neonatal period) as the reference category. We estimated two models. First, we fit a model of mortality outcome on skilled attendance at birth, adjusting for the control variables described above. Second, we fit a model of mortality outcome on number of ANC interventions received, and the immediate postnatal variables controlling for socio–demographic variables. The latter model included data from 18 countries and was restricted to only births delivered with skilled health personnel to assess the effect of quality of care on newborn death. The mortality computation analyses were carried out in STATA version 13 while the regression models were implemented in SAS.

## RESULTS

**Figure 1:**
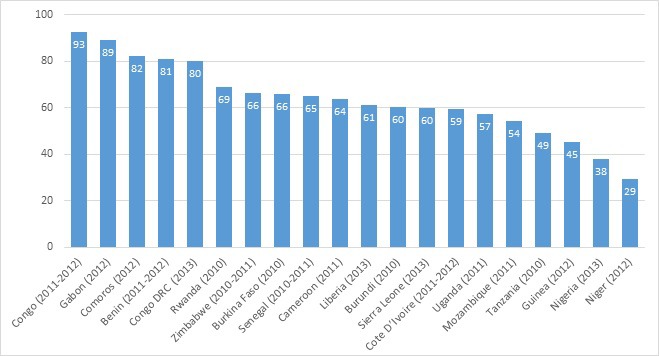
Percent of live birth in the five years preceding the survey with skilled attendant at birth by country, Demographic and Health Survey (DHS, 2010–2014). Survey years are included in the parenthesis.

**Figure 2:**
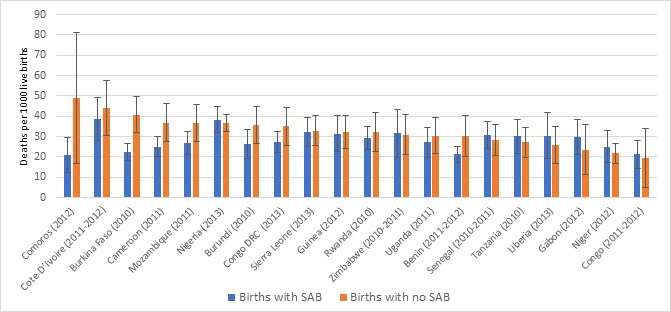
Neonatal mortality rate and 95% confidence intervals according to whether the birth had a skilled attendant at birth (SAB) or not by country.

**Table 1** and **Figure 1** show levels of coverage of skilled attendant at birth (SAB) by country. Coverage ranged from 29% in Niger to 93% in Congo with a median of 62%. Table 1 also shows the five–year neonatal mortality rate by country, ranging from 22 deaths per 1000 live births in Congo to 39 deaths per 1000 live births in Sierra Leone. There is a marginally significant inverse relationship between SAB and neonatal mortality: (r = –0.42, *P* < 0.0619). While Congo shows the highest coverage of SAB and lowest neonatal mortality, the country with lowest coverage of SAB (Niger) does not have the highest neonatal mortality. **Figure 2** presents the neonatal mortality rate by SAB along with the 95% confidence intervals. The general picture across countries suggests no significant survival advantage during the neonatal period among births with SAB and those without. Based on the confidence intervals, there is no statistically significant difference in neonatal mortality rate among births with SAB compared to those without, except in Burkina Faso where births with SAB have significantly lower neonatal mortality than those without.

**Table 2** presents results from the multinomial mixed model regression. Births who survived beyond the neonatal period are used as reference category. Furthermore, the reference category corresponding to each categorical variable included in the model is shown in parenthesis beside the name of the variable in the first column. Column 2 shows the age at death, distinguishing deaths on days 0–1 and deaths on days 2–27, and column 3 shows the response category of the independent variable included in the model. Response categories with significant results are bolded. Adjusting for covariates in the model, there is no statistically significant difference in the odds of death on days 0–1 between births with SAB and births without. However, births with SAB who survived after the first day showed a statistically significant 16% lower risk of death on the period 0–27 days compared to births without SAB (OR = 0.84, 95% CI = 0709–0.996). Several demographic covariates remained statistically significant in the model. These include parity, mother’s age at birth of the child, and current marital status. With regard to parity, there is no differential risk of death for first birth compared to births of parity 5 plus whether on days 0–1 or days 2–27. However, births of parity 2–4 have significantly 25% lower odds of death on the days 2–27 compared to births of parity 5 plus. Compared to births to women aged 20–29, births to older women have higher odds of death on either days 0–1 or days 2–27. Births to women aged 40 or more have respectively 72% and 62% higher odds of death on days 0–1 and days 2–27 compared to births to women aged 20–29. For births to women 30–39, the differential risk is observed only for births on days 0–1, with a 25% increased odds of death. Births to single women have 52% higher odds of death compared to births to married women. Other characteristics such as education level, residence or wealth quintile showed no significant differential risk of death on days 0–1 or days 2–27.

**Table 2:** Adjusted odds ratios of death on days 0–1 or days 2–27 compared to surviving over the neonatal period among births with SAB compared to births without SAB)*

Variable (reference category)	Age at death	Category	Odds Ratio	95% confidence interval		*P*
Skilled attendant at birth (No.)	Day 0–1	Yes	1.17	0.934	1.465	0.173
	**Day 2–27**	**Yes**	**0.84**	**0.709**	**0.996**	**0.045**
Parity (5 plus)	Day 0–1	1	1.35	0.919	1.984	0.127
	Day 2–27	1	1.03	0.708	1.506	0.870
	Day 0–1	2–4	0.84	0.678	1.051	0.129
	**Day 2–27**	**2–4**	**0.75**	**0.607**	**0.931**	**0.009**
Mother's age at birth (20–29)	Day 0–1	15–19	0.91	0.757	1.106	0.358
	Day 2–27	15–19	1.15	0.879	1.517	0.301
	**Day 0–1**	**30–39**	**1.25**	**1.052**	**1.488**	**0.011**
	Day 2–27	30–39	1.14	0.937	1.376	0.194
	**Day 0–1**	**≥40**	**1.72**	**1.349**	**2.187**	**<0.0001**
	**Day 2–27**	**≥40**	**1.62**	**1.247**	**2.099**	**<0.0001**
Current marital status (Married)	Day 0–1	Other	1.01	0.871	1.172	0.896
	Day 2–27	Other	1.15	0.939	1.414	0.175
	Day 0–1	Single	1.08	0.811	1.427	0.611
	**Day 2–27**	**Single**	**1.52**	**1.218**	**1.906**	**<0.0001**
Education level (No education)	Day 0–1	Primary	1.04	0.896	1.215	0.585
	Day 2–27	Primary	1.04	0.92	1.171	0.545
	Day 0–1	Secondary or higher	0.92	0.704	1.202	0.542
	Day 2–27	Secondary or higher	0.84	0.649	1.089	0.189
Residence (Rural)	Day 0–1	Urban	1.01	0.837	1.218	0.920
	Day 2–27	Urban	1.14	0.92	1.401	0.237
Wealth quintile (Poorest)	Day 0–1	Poorer	1.03	0.852	1.239	0.776
	Day 2–27	Poorer	1.05	0.893	1.241	0.542
	Day 0–1	Middle	1.04	0.911	1.180	0.585
	Day 2–27	Middle	1.00	0.805	1.234	0.973
	Day 0–1	Richer	1.04	0.877	1.224	0.678
	Day 2–27	Richer	0.94	0.755	1.173	0.590
	Day 0-1	Richest	1.05	0.828	1.323	0.705
	Day 2-27	Richest	0.91	0.619	1.328	0.614

SAB – skilled attendant at birth

*Analysis based on generalized linear mixed model; pooled DHS 2010-2014 from 20 countries in Africa.

In **Table 3**, we present results from the multinomial mixed model regression, restricted to only births with SAB and assessing the effect of co–coverage of interventions during ANC and immediate postnatal interventions, adjusting for the same socio–demographic characteristics. Statistically significant variables are shown in bold. Adjusting for the socio–demographic variables included in the model and immediate postnatal interventions, there is a strong and negative significant association between the number of ANC interventions received and the odds of death on days 2–27. The odds of death on days 2–27 are reduced by 5% for each additional ANC intervention received. However, this advantage was not observed on days 0–1 and in fact there appeared to be a marginal positive association between number of ANC interventions and risk of death. Regarding immediate postnatal interventions, newborns weighed at birth or those initiated early on breastfeeding were significantly less likely to die either on days 0–1 or days 2–27. Newborns weighed at birth were 58% less like die on days 0–1 and 40% less likely to die on days 2–27. Similarly, newborns initiated early on breastfeeding were respectively 76% and 41% less likely to die on days 0–1 and days 2–27. The demographic characteristics such as parity, mother’s age and marital status remained significant and in the similar direction as described for the model in **Table 2**. However, urban–rural residence, education level and wealth quintile became statistically significant in this model in somewhat unexpected direction.

**Table 3:** Adjusted odds ratios of death on days 0–1 or days 2–27 compared to surviving over the neonatal period by number of ANC interventions received by mother and immediate postnatal interventions received by newborn (among births with SAB)*

Independent variable	Age at death	Category	Odds ratio	95% confidence interval		P–value
**Number of seven ANC interventions received by mother**	**Day 0–1**	**Continuous**	**1.02**	**1.000**	**1.044**	**0.0545**
	**Day 2–27**	**Continuous**	**0.95**	**0.927**	**0.979**	**0.0005**
Newborn weighed at birth (No.)	**Day 0–1**	**Yes**	**0.42**	**0.292**	**0.615**	**<.0001**
	**Day 2–27**	**Yes**	**0.60**	**0.450**	**0.809**	**0.0007**
Newborn initiated early on breastfeeding (No)	**Day 0–1**	**Yes**	**0.24**	**0.184**	**0.310**	**<.0001**
	**Day 2-27**	**Yes**	**0.59**	**0.473**	**0.744**	**<.0001**
Parity (5 plus)	**Day 0-1**	**1**	**1.54**	**1.030**	**2.307**	**0.0356**
	Day 2-27	1	1.19	0.817	1.728	0.3668
	Day 0-1	2–4	0.91	0.723	1.151	0.4372
	**Day 2-27**	**2**–**4**	**0.80**	**0.645**	**1.002**	**0.0518**
Mother's age at birth (20-29)	Day 0-1	15-–19	0.86	0.702	1.043	0.1225
	Day 2-27	15–19	1.04	0.790	1.361	0.7941
	**Day 0-1**	**30**–**39**	**1.27**	**1.056**	**1.519**	**0.011**
	Day 2-27	30–39	1.16	0.941	1.432	0.1633
	**Day 0-1**	≥**40**	**1.80**	**1.376**	**2.364**	**<.0001**
	**Day 2–27**	≥**40**	**1.60**	**1.192**	**2.142**	**0.0017**
Current marital status (Married)	Day 0–1	Other	1.02	0.874	1.179	0.8458
	Day 2–27	Other	1.09	0.898	1.318	0.3867
	Day 0–1	Single	1.05	0.799	1.389	0.7119
	**Day 2–27**	**Single**	**1.47**	**1.162**	**1.861**	**0.0013**
Education level (No education)	**Day 0–1**	**Primary**	**1.23**	**1.064**	**1.417**	**0.0051**
	Day 2–27	**Primary**	**1.14**	**0.990**	**1.304**	**0.0695**
	Day 0–1	Secondary or higher	1.17	0.883	1.549	0.2734
	Day 2–27	Secondary or higher	0.97	0.770	1.215	0.7736
Residence (Rural)	Day 0–1	Urban	1.13	0.917	1.401	0.2456
	**Day 2–27**	**Urban**	**1.27**	**1.030**	**1.559**	**0.0254**
Wealth quintile (Poorest)	Day 0–1	Poorer	1.14	0.937	1.378	0.1945
	Day 2–27	Poorer	1.11	0.944	1.304	0.2079
	**Day 0–1**	**Middle**	**1.27**	**1.096**	**1.463**	**0.0014**
	Day 2–27	Middle	1.08	0.888	1.323	0.4300
	**Day 0–1**	**Richer**	**1.42**	**1.155**	**1.736**	**0.0008**
	Day 2–27	Richer	1.07	0.850	1.338	0.5777
	**Day 0–1**	**Richest**	**1.68**	**1.267**	**2.225**	**0.0003**
	Day 2–27	Richest	1.08	0.767	1.527	0.6531

ANC – antenatal care, SAB – skilled attendant at birth

Analysis based on generalized linear mixed model; pooled DHS 2010–2014 data from 18 countries in Africa.

## DISCUSSION

Access to skilled attendant at birth during antenatal care and delivery is promoted as a key strategy for improving maternal and newborn care in low and middle–income countries. The importance of skilled personnel at the time of birth is widely acknowledged, such that the proportion of births attended by skilled health personnel has been adopted as a key coverage monitoring indicator for the Sustainable Development Goal 3.1. However, we showed in this study that the survival benefits expected for newborns delivered with skilled health personnel are not being observed in sub–Saharan Africa. We demonstrated in the analysis that there is no survival benefit on days 0–1 for newborns, whether they are delivered by a skilled birth attendant or not. Only when they survive day 1 does such benefit occur. These results are consistent with previous studies and call once again for greater attention to the fragility of care around the time around of delivery [3,4,11]. The results suggest that skilled birth attendants, and most health facilities, are not yet equipped enough to save newborns at highest risk of death immediately after birth. These results were further corroborated by assessing effects of quality of care immediately after birth on the risk of death among births with skilled attendant. Among these births, simple interventions such as being weighed at birth or being initiated on breastfeeding early showed strong and significant benefits for survival chances during the neonatal period. It may be that these interventions are also good proxy for quality of care during the immediate postnatal period. Using the number of antenatal care interventions that the mother received did not show survival benefits for births attended by skilled personnel on days 0–1, but only on days 2–27, reinforcing the finding that interventions delivered immediately after birth are most critical for the survival of the newborn during the immediate periods following birth.

In **Table 4**, we estimated, based on annual births, stillbirth and number of skilled health professionals, the number of deliveries per skilled health professional given current coverage, and for 100% coverage. The estimated number of deliveries per skilled health professional ranges from 8 in Nigeria to 120 in Guinea. While these are average estimates and do not account for highly unequal distribution of skilled health professional within countries or that some health professional may not conduct deliveries at all, and there is highly unequal distribution of deliveries for each provider, their level is not extremely high as to overwhelm the health system. Lack of significantly improved survival among those who access these professionals during the immediate postnatal period may suggest that the system is not sufficiently equipped and strong enough to deal with high–risk obstetric conditions and/or a substantial portion of these health personnel is not skilled enough to prevent such risks from leading to death. It also suggests highly inequitable distribution of SAB or facilities with lower access in higher risk populations. Even with 100% coverage of skilled delivery, a country like Nigeria will see only an average of 20 deliveries per skilled health personnel, and in most countries skilled health personnel will perform on average fewer than 100 deliveries per year.

**Table 4:** Estimated average annual deliveries per skilled health professional by country

Country	Total population 2015 (in 1000s)^a^	Births in 2015 (in 1000s)*	Number of stillbirths in 2015**†**	Skilled health professionals density (per 10 000 population)**‡**	Estimated No. of skilled health professionals	Percentage of births with skilled health professional	Number of annual deliveries per skilled health professional**§**	Number of annual deliveries per skilled health professional if 100% coverage
Benin	10 880	388	11 700	8.3	9030	80.9	36	44
Burkina Faso	18 106	717	14 900	6.1	11 044	65.9	44	66
Burundi	11 179	488	12 700	–	–	60.3	–	–
Cameroon	23 344	847	16 400	5.2	12 139	63.6	45	71
Comoros	788	26	800	–	–	82.2	–	–
Congo	4620	167	2500	9.2	4251	92.5	37	40
Cote d’Ivoire	22 702	838	22 800	6.3	14 302	59.4	36	60
Democratic Republic of the Congo	77 267	3217		–	–	80.1	–	–
Gabon	1725	51	700	–	–	89.3	–	–
Guinea	12609	460	9900	1.4	1765	45.3	120	266
Liberia	4503	156	3300	2.9	1306	61.1	75	122
Mozambique	27 978	1087	20 700	4.5	12 590	54.3	48	88
Niger	19 899	983	36 200	1.6	3184	29.3	94	320
Nigeria	182 202	7133	313 700	20.1		38.1	8	20
Rwanda	11 610	363	5900	7.5	8707	69.0	29	42
Senegal	15 129	567	14 500	4.8	7262	65.1	52	80
Sierra Leone	6453	229	5400	1.9	1226	59.7	114	191
Uganda	39 032	1665	34 200	14.2	55 426	57.4	18	31
United Republic of Tanzania	53 470	2064	47 100	4.7	25 131	48.9	41	84
Zimbabwe	15 603	539		14.2	22 156	66.2	16	25

*Source: United Nations, Department of Economic and Social Affairs, Population Division [22].

†Source: Lawn et al [23].

‡Source: WHO [24].

§Assumed same coverage level of skilled health personnel for stillbirths.

In such conditions, it is not entirely inappropriate to question whether it still makes sense to continue to advocate for increased skilled delivery when this strategy is not producing the expected survival advantage in resource constrained countries. The answer from our study would be yes, because, at least beyond the first two days of life, there is significant survival advantage for newborns delivered with skilled birth attendants. Nevertheless, the question underscores the tremendous missed opportunities for the health system when women are encouraged to deliver in health facilities with skilled attendant, yet do not receive needed quality of care when they show up. The lack of survival advantage during the first days of life for the newborn, even when delivery occur with skilled personnel or in health facility can reinforce barriers to facility use.

Our results raise three main implications, which are also generally raised to explain the lack of significant improvement in survival of newborns delivered with skilled attendants compared those without. First is the need for improving access to equitable high–quality maternal and delivery care in African countries. Many studies have demonstrated the low level of quality of care, including low access to basic and emergency obstetric services. Nesbitt and colleagues showed a quality gap, defined as the difference between the crude coverage of SAB and the coverage of SAB with high quality of care, as large as 50 percentage points in health facilities in several districts in Ghana. Marchant raised the concern that contacts with the health system are not used sufficiently to deliver life–saving, timely interventions. While these studies have either assessed readiness of health facilities or delivery of interventions according to international or WHO–recommended standards, another important knowledge gap remains, namely an accurate assessment of care provision decision–making process based on obstetric risk to the woman or her newborn in resource–constrained settings. In an environment where there are not enough equipment, drugs or human resources, how health professionals decide on who should get what interventions and who should not, is not always factored in the measure of quality of care. It is clear that if, given such shortages, only a few patients can receive an intervention, some triage process will need to be in place based on obstetric risk. Under such circumstances, measures of quality of care based on optimal international standards applicable to every woman regardless of risk, or a sum of required interventions that every pregnant woman must receive will always yield low quality of care. The understanding of care provision and triage decision–making process that health care workers are forced to make when faced with a shortage of essential equipment and drugs will allow an appropriate and contextualized remedy on how countries should adapt international standards to their specific resource constrained contexts. For example, to tackle the shortage of qualified health professionals, countries have resorted to task–shifting, with increasing reliance on lower level health professionals, including in some cases community health workers. However, it is essential that countries which adopt such strategies ensure that it is accompanied with the appropriate education, skill upgrade training, and necessary equipment within a mentorship and supportive environment. Another critical aspect is the extent to which quality of care is equitable across facilities, regions and population groups. Equitable access to quality care is closely linked to population level effective coverage, a proximal determinant of survival impact. In a context of equitable access, results shown in **Table 4** would not imply overburdened delivery system, in terms of human resources.

Second, while there is a standard definition of skilled health personnel, its application at country level and its measurement remain a challenge at country level [5]. While WHO and UNICEF are rallying the midwifery and newborn communities to revise the current definition, its applicability at country level will always face contextual challenges, where most of qualified health personnel do not work where the need for them is highest. Rural, poor and difficult access areas will remain disadvantaged since no doctors or qualified personnel will opt to go there without substantial benefits. Countries will therefore continue to use task shifting strategy to address accessibility issues in poor areas. Under such conditions, expanding the training of all those involved in maternal and newborn care to raise their skills, and equipping facilities for basic and emergency obstetric and newborn care would be a reasonable strategy. Furthermore, the measurement of skilled birth attendant through household surveys is also challenged with misclassification and inaccuracy. Low literacy mothers delivering in facilities do not always know the cadre of health professional in charge of their delivery [25,26]. In addition, categories of cadres of health personnel used in survey instruments such as those in DHS or Multiple Indicator Cluster Surveys (MICS) are not consistent across time and countries. Furthermore, the type of cadres that are included as skilled varies largely within and between countries. This creates difficulties in assessing accurately, not only the coverage levels of SAB but its trends. A growing number of studies are now researching measurement approaches based on linking of household surveys to health facilities [27,28]. While findings from these studies will be very valuable, an immediate step to take is for large survey programs such as the DHS and the Multiple Indicator Cluster Surveys (MICS) to standardize their instruments across surveys and time to allow comparability of results over time. While measurement issues with skilled attendant at birth could have affected our findings above, it is important to note that the large majority of births reported being delivered with skilled personnel occurred in health facilities in these countries. The findings therefore stand for births occurring in health facilities and highlight the tremendous missed opportunities and insufficient quality of care that substantial number of pregnant women face in countries included in this study.

Finally, there has been a suggestion that lack of improved outcomes at facility levels could be due to higher obstetric risk pregnancies rushing to the facility, which increases the risk profile of births with skilled attendants vs those without [16–18]. While this may be happening, it also suggests that drastic measures to improve access to timely antenatal care, counselling on institutional delivery or access to skilled delivery and attention to quality of care, including respectful maternal care, are to be prioritized. Implementation of quality pre–delivery maternity homes may be a solution to delayed access in some contexts [29].

Ending preventable newborn death while relying on current health systems in sub–Saharan African countries will require not only improvement in access to skilled delivery but also drastic measures to ensure effective coverage by improving availability of equipment and essential medicine, and equitable distribution of health personnel that is ready to deliver lifesaving interventions especially at time around delivery.

## References

[1] You D, Hug L, Ejdemyr S, Idele P, Hogan D, Mathers C (2015). Global, regional, and national levels and trends in under-5 mortality between 1990 and 2015, with scenario-based projections to 2030: a systematic analysis by the UN Inter-agency Group for Child Mortality Estimation.. Lancet.

[2] UNICEF. WHO. Every Newborn: An action plan to end preventable newborn deaths. Geneva: World Health Organization; 2014.

[3] Bhutta ZA, Das JK, Bahl R, Lawn JE, Salam RA, Paul VK (2014). Can available interventions end preventable deaths in mothers, newborn babies, and stillbirths, and at what cost?. Lancet.

[4] Lawn JE, Blencowe H, Oza S, You D, Lee ACC, Waiswa P (2014). Every Newborn: progress, priorities, and potential beyond survival.. Lancet.

[5] World Health Organization. Making pregnancy safer: the critical role of the skilled attendant: a joint statement by WHO, ICM and FIGO. Geneva: World Health Organization; 2004.

[6] World Health Organization. Competent attendance in maternal and newborn health: the definition of the competent health care provider in maternal and newborn health. A joint statement by WHO, UNFPA, UNICEF, ICM, ICN, FIGO and IPA. Available: http://www.who.int/reproductivehealth/skilled-birth-attendant/en/. Accessed: 19 September 2017.

[7] United Nations. The Millennium Development Goals Report 2015. Available: http://www.un.org/millenniumgoals/2015_MDG_Report/pdf/MDG%202015%20rev%20(July%201).pdf. Accessed: 19 September 2017.

[8] World Health Organization. Strategies toward ending preventable maternal mortality (EPMM). Geneva: World Health Organization; 2015.

[9] Nesbitt RC, Lohela TJ, Manu A, Vesel L, Okyere E, Edmond K (2015). Quality along the Continuum: A Health Facility Assessment of Intrapartum and Postnatal Care in Ghana.. PLoS One.

[10] Marchant T, Tilley-Gyado RD, Tessema T, Singh K, Gautham M, Umar N (2015). Adding Content to Contacts: Measurement of High Quality Contacts for Maternal and Newborn Health in Ethiopia, North East Nigeria, and Uttar Pradesh, India.. PLoS One.

[11] Hodgins S, D’Agostino A (2014). The quality–coverage gap in antenatal care: toward better measurement of effective coverage.. Glob Health Sci Pract.

[12] Singh K, Brodish P, Suchindran C (2014). A Regional Multilevel Analysis: Can Skilled Birth Attendants Uniformly Decrease Neonatal Mortality?. Matern Child Health J.

[13] Dickson KE, Kinney MV, Moxon SG, Ashton J, Zaka N, Simen-Kapeu A (2015). Scaling up quality care for mothers and newborns around the time of birth: an overview of methods and analyses of intervention-specific bottlenecks and solutions.. BMC Pregnancy Childbirth.

[14] Sharma G, Mathai M, Dickson KE, Weeks A, Hofmeyr GJ, Lavender T (2015). Quality care during labour and birth: a multicountry analysis of health system bottlenecks and potential solutions.. BMC Pregnancy Childbirth.

[15] Blanc AK, Warren C, McCarthy KJ, Kimani J, Ndwiga C (2016). RamaRao S. Assessing the validity of indicators of the quality of maternal and newborn health care in Kenya.. J Glob Health.

[16] Paul BK, Rumsey DJ (2002). Utilization of health facilities and trained birth attendants for childbirth in rural Bangladesh: An empirical study.. Soc Sci Med.

[17] Ronsmans C, Chowdhury ME, Koblinsky M, Ahmed A (2010). Care seeking at time of childbirth, and maternal and perinatal mortality in Matlab, Bangladesh.. Bull World Health Organ.

[18] Ronsmans C Scott S Qomariyah SN Achadi E Braunholz D Marshall Tet alMidwife-led community care and maternal mortality in Indonesia. Bull World Health Organ 2009 87 416 23 10.2471/BLT.08.051581 19565119 PMC2686212

[19] Demographic and health surveys. Available: https://www.dhsprogram.com/. Accessed: 18 October 2017.

[20] Preston SH, Heuveline P, Guillot M. Demography: Measuring and modeling population processes. Oxford: Blackwell Publishers; 2001.

[21] Lohr SL. Sampling: design and analysis. Boston: Cengage Learning; 2010.

[22] United Nations, Department of Economic and Social Affairs, Population Division. World Population Prospects: The 2015 Revision. New York: UN; 2015.

[23] Lawn JE, Blencowe H, Waiswa P, Amouzou A, Mathers C, Hogan D (2016). Stillbirths: rates, risk factors, and acceleration towards 2030.. Lancet.

[24] WHO. World Health Statistics 2016. Monitoring Health for the SDGs. Geneva: WHO; 2016.

[25] Blanc AK, Warren C, McCarthy KJ, Kimani J, Ndwiga C (2016). RamaRao S. Assessing the validity of indicators of the quality of maternal and newborn health care in Kenya.. J Glob Health.

[26] McCarthy KJ, Blanc AK, Warren C, Kimani J, Mdawida B, Ndwidga C (2016). Can surveys of women accurately track indicators of maternal and newborn care? A validity and reliability study in Kenya.. J Glob Health.

[27] Do M, Micah A, Brondi L, Campbell H, Marchant T, Eisele T (2016). Linking household and facility data for better coverage measures in reproductive, maternal, newborn, and child health care: Systematic review.. J Glob Health.

[28] Carvajal-Aguirre L Mehra V Amouzou A Khan SM Vaz L Guenther T Does health facility service environment matter for the receipt of essential newborn care? Linking health facility and household survey data in Malawi. J Glob Health 201776 020509 10.7189/jogh.07.020509 PMC5804506 29423185

[29] van Lonkhuijzen L, Stekelenburg J, van Roosmalen J (2009). Maternity waiting facilities for improving maternal and neonatal outcome in low-resource countries.. Cochrane Database Syst Rev.

